# Structure–Biomedical Activity Relationship of Tunable Ceria–Graphene Nanocomposites Leading to Divergent Cellular Responses

**DOI:** 10.3390/ijms27114772

**Published:** 2026-05-26

**Authors:** Tudor-Mihai Magdaș, Ioana Bâldea, Constantin Bodolea, Andrei Mihai Bălan, Adrian Ștef, Lidia Mǎgeruşan, Gabriela Adriana Filip

**Affiliations:** 1 Anesthesia and Intensive Care Department, “Iuliu Hatieganu” University of Medicine and Pharmacy, 8 Victor Babeş Street, 400012 Cluj-Napoca, Romania; tudor.miha.magdas@elearn.umfcluj.ro (T.-M.M.); constantin.bodolea@umfcluj.ro (C.B.); balan.andrei@umfcluj.ro (A.M.B.); stef.adrian@yahoo.com (A.Ș.); 2 National Institute for Research and Development of Isotopic and Molecular Technologies, 67-103 Donat Street, 400293 Cluj-Napoca, Romania; gabriela.filip@umfcluj.ro; 3 Department of Anesthesia and Intensive Care, Heart Institute “Niculae Stancioiu”, “Iuliu Hatieganu” University of Medicine and Pharmacy, 19-21 Motilor Street, 400001 Cluj-Napoca, Romania; 4 Department of Physiology, Faculty of Medicine, University of Medicine and Pharmacy “Iuliu Hatieganu”, 400347 Cluj-Napoca, Romania; ioana.baldea@umfcluj.ro; 5 Department of Anesthesia and Intensive Care, Municipal Clinical Hospital, 400139 Cluj-Napoca, Romania; 6 Department of Anatomy and Embryology, “Iuliu Hațieganu” University of Medicine and Pharmacy, 8 Victor Babes Street, 400012 Cluj-Napoca, Romania

**Keywords:** cerium-containing graphenes, graphene-based nanomaterials, electrochemical exfoliation, structure-activity relationship, hormesis, selective cytotoxicity

## Abstract

Graphene-based nanomaterials (GBNs) have emerged as promising candidates for diverse biomedical applications, but their clinical translation has been hindered by inherent cytotoxicity. We synthesized three distinct cerium-containing graphene nanocomposites using a single-step, in situ electrochemical exfoliation process and investigated their structure–activity relationships in normal dermal fibroblasts (BJ) and hepatocarcinoma cells (HepG2). The properties of the resulting nanocomposites, including their morphology, cerium loading, and the surface redox state (Ce^3+^/Ce^4+^ ratio) were directly dictated by the employed synthesis parameters, such as the cerium salt precursor and its concentration. These distinct materials induced differential cellular responses that ranged from preferential cytotoxicity in HepG2 cells to a significant cytostimulatory effect and increased ATP levels in BJ fibroblasts, particularly in EXF3-treated cells. Our findings indicate that by employing the in situ electrochemical exfoliation method, the hybrid graphene compounds might be further tailored for specific purposes, moving the narrative beyond the mere functionalization of the graphene in order to achieve biocompatibility.

## 1. Introduction

Graphene-based nanomaterials (GBNs) are a class of two-dimensional materials composed of single- or few-layered sheets of *sp*^2^-hybridized carbon atoms arranged in a hexagonal lattice [1,2,3,4]. Their unique structure offers excellent physicochemical properties, making GBNs promising candidates for a wide array of biomedical applications [5,6,7,8]. The large surface area enables controlled high-capacity loading via π–π stacking, hydrogen bonding, and hydrophobic interactions, which, paired with their capacity to easily cross cell membranes, highlights them as ideal nanocarriers for controlled drug release and gene transfection [5,9,10,11,12,13]. The inherent optical properties of the graphene derivates, such as nano-graphene oxide (nGO) and graphene quantum dots (GQDs), possess intrinsic photoluminescence and a strong absorbance in the near-infrared range (NIR), which is applied in bioimaging and photothermal therapy [14,15,16]. Due to their remarkable mechanical strength, graphene-based materials are used in tissue engineering as reinforcing fillers to improve the scaffolds’ stability [17,18,19]. However, clinical translation of GBNs has been limited by their intrinsic cytotoxicity. Experimental in vitro studies demonstrated that graphenes induce a dose-dependent cytotoxicity [6], mediated through multiple mechanisms, including direct physical damage [20,21], the generation of excessive reactive oxygen species (ROS) and subsequent oxidative stress, inflammatory pathways activation, mitochondrial damage, and apoptosis [6,22,23]. More concerning, these findings were validated in in vivo studies, which revealed that GBNs accumulate in the reticuloendothelial system (e.g., liver, spleen) and exert systemic toxicity [24,25,26,27,28]. In humans, occupational exposure has led to severe lung disorders by inhalation of aerosolized graphenes [29,30,31].

Surface functionalization of the graphene scaffold with organic or inorganic materials is an efficient method to reduce their intrinsic toxicity [2,7,32,33]. Ceria nanoparticles (CeO_2_-NPs) emerged as a promising candidate for surface functionalization, due to their remarkable redox properties [34,35]. By reversibly shifting between the Ce^3+^ and Ce^4+^ oxidation states, CeO_2_-NPs can limit the cellular damage induced by reactive oxygen species (ROS) by mimicking the activity of antioxidant enzymes like the superoxide dismutase (SOD) and catalase (CAT) and subsequently reduce inflammation [36,37,38,39]. The antioxidant properties are particularly relevant in medical conditions where chronic inflammation plays a central role in the pathophysiological processes, including neurodegenerative diseases and retinal damage, where CeO_2_ nanoparticles have attenuated disease progression [40,41]. However, these nanoparticles can also exhibit cytotoxic effects under certain conditions, enabling their safe use within a therapeutic window [33].

Limited data from prior in vitro experimental studies indicate that graphene oxide (GO) or reduced graphene oxide (rGO) functionalized with ceria nanoparticles possess high biocompatibility in normal cell lines while being highly toxic to cancer cell lines, a property thought to be related to the acidic tumor microenvironment [8,42,43,44]. These prior studies rely on materials obtained in multi-step ex situ functionalization methods, where pre-synthesized graphene derivatives are decorated with nanoparticles. This approach may constrain the overall performance, due to limited control over the graphene’s structure. To overcome these drawbacks, this study employed a novel in situ electrochemical exfoliation technique for developing graphene-cerium-containing nanohybrid materials, in which the graphene layers are simultaneously formed and functionalized. This environmentally friendly and straightforward approach offered a direct pathway in which both graphite exfoliation and the incorporation of cerium species occur simultaneously within a single step, at room temperature, avoiding the use of organic solvents or harsh reduction chemicals that are typically employed for chemical synthesis. Furthermore, the electrochemical route presents several advantages over conventional preparation methods since the obtained material’s physicochemical properties, including cerium loading, can be easily tuned by altering the nature and the concentration of the precursor salts employed as electrolytic medium, the value of applied voltage, and the reaction time, overcoming the limitations of multi-step synthesis procedures.

The current study aims to investigate the effect of various cerium-functionalized graphene-based nanomaterials in normal dermal fibroblasts (BJ) and cancerous hepatocarcinoma (HepG2) cell lines. For this reason we evaluated the biocompatibility and the structure–activity relationship of three cerium-functionalized graphene-based nanomaterial formulations obtained through liquid-phase graphite exfoliation in different cerium-containing salt solutions, each of them with different physicochemical properties. Cerium nitrate and cerium sulfate were selected as chemical precursors for the preparation of the electrolytic exfoliation medium because they provide different counter-ions and oxidation environments during electrochemical exfoliation, which can influence cerium incorporation and nanoparticle nucleation on graphene sheets, while different concentrations (0.2 M and 0.1 M) were employed to modulate the nanoparticle density and the resulting surface redox state (Ce^3+^/Ce^4+^ ratio). This design allowed for a systematic evaluation of how these structural parameters influence bioactivity.

The nanocompounds were comprehensively characterized, as properties like surface chemistry, aggregation state and porosity are reported to strongly influence the cellular interactions and ultimately the cytotoxic profile of the compound [3,32,33].

## 2. Results

### 2.1. Characterization of Cerium-Doped Graphene-Based Nanomaterials

Three types of cerium-containing graphene-based nanomaterials were synthesized employing different cerium salt precursors at different concentrations (Ce(NO_3_)_3_·6H_2_O for EXF1 at 0.2 M; Ce(SO_4_)_2_ at 0.2 M for EXF2 and 0.1 M for EXF3) as electrolytic media. The resulting hybrid nanocomposites were analyzed to assess their morphological and structural characteristics.

**Figure 1:**
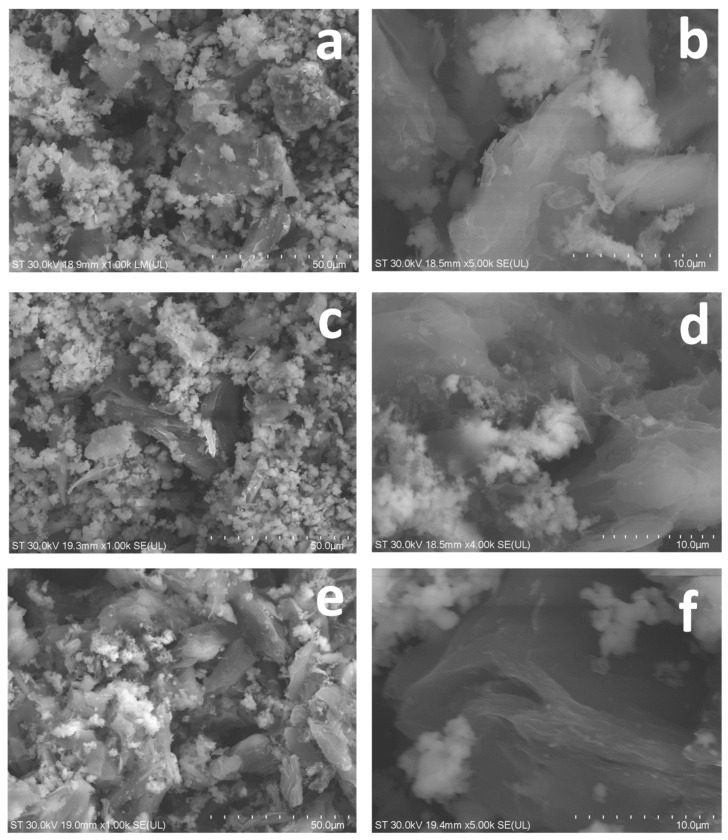
STEM analysis of EXF1 ((**a**) scale bar 50 µm, (**b**) scale bar 10 µm); EXF2 ((**c**)—scale bar 50 µm, (**d**) scale bar 10 µm); and EXF3 ((**e**) scale bar 50 µm, (**f**) scale bar 10 µm).

**Figure 2:**
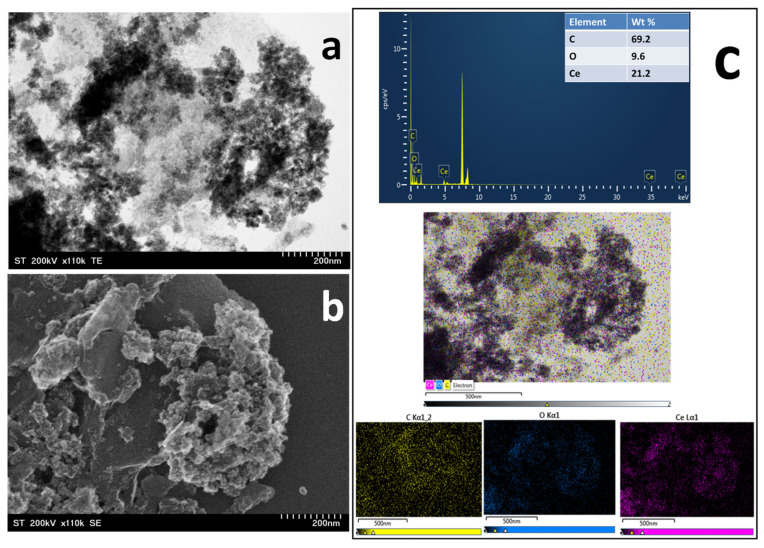

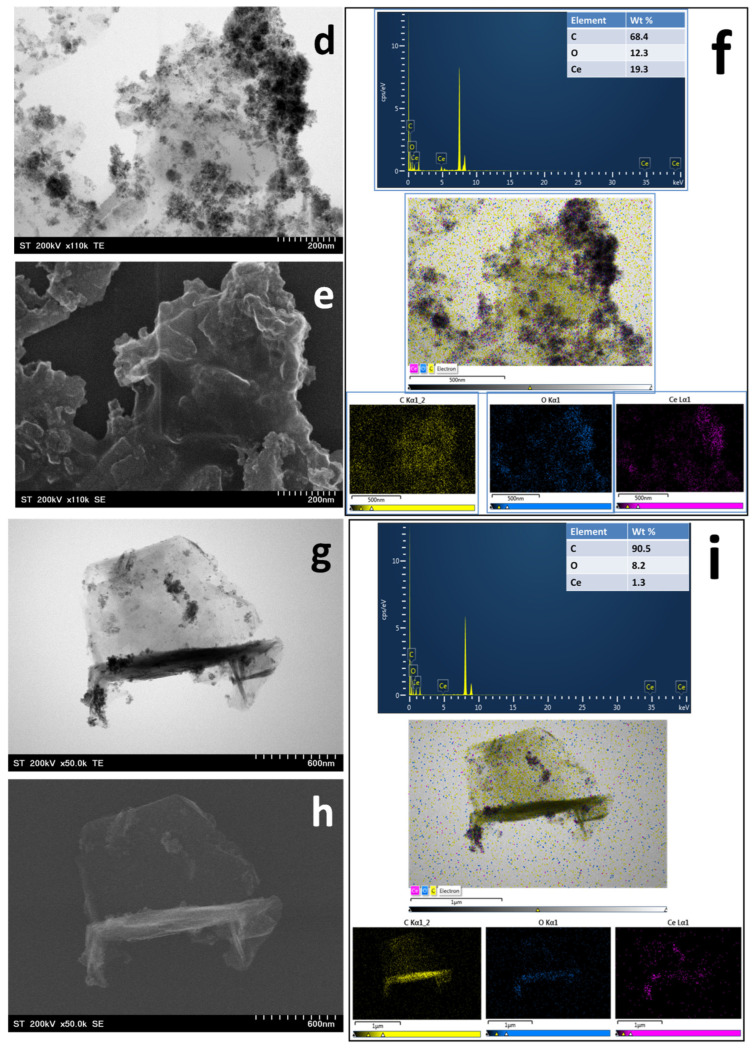
TEM ((**a**) scale bar 200 nm), SEM ((**b**) scale bar 200 nm), and SEM-EDX elemental mapping of C, O, and Ce atoms (**c**) in case of EXF1; TEM ((**d**) scale bar 200 nm), SEM ((**e**) scale bar 200 nm) and SEM-EDX elemental mapping of C, O, and Ce atoms (**f**) in EXF2; TEM ((**g**) scale bar 600 nm), SEM ((**h**) scale bar 600 nm), and STEM-EDX elemental mapping of C, O, and Ce atoms (**i**) in case of EXF3.

Key morphological features are exposed using STEM analysis. Figure 1 shows the characteristic layered structure of graphene-based materials and confirms the wrinkled and folded texture featuring rough surfaces. The cerium presence is indicated by the existence of white spots distributed at the surface decorating the graphene sheets. In EXF1 (Figure 1a,b) and EXF2 (Figure 1c,d), numerous bright spots and clusters are visible, corresponding to the cerium aggregates decorating the wrinkled graphene sheets. Consistent with the TEM and EDX data (Figure 2), the density of these bright cerium clusters is notably reduced in the structure of EXF3 (Figure 1e,f), which features smoother, less decorated graphene surfaces due to the employed preparation conditions and the concentration of the cerium-containing chemical precursor. Note that while the fundamental structure is determined by nanoscale graphene sheets adorned with cerium oxide domains, any micron-sized aggregates are the consequence of restacking events typical for graphene-based materials.

Further, TEM and SEM investigations provided clear insights into the morphology and the impact of synthesis parameters on the resulting materials. In all samples, the presence of exfoliated graphene is evident, appearing as large-dimension, thin, clear profiles, with wrinkled and crumpled surface and plenty of cerium-containing nanoparticles represented by the dark spots that decorate the graphene sheets. However, the doping degree varied significantly based on the employed synthesis conditions. EXF1 (Figure 2a,b), synthesized using 0.2 M cerium nitrate solution, exhibits the highest loading density. The graphene sheets are heavily decorated by dense aggregates of nanoparticles, resulting in a rough surface texture where the underlying scaffold is partially obscured. EXF2 (Figure 2d,e), synthesized using 0.2 M cerium sulphate solution, also shows significant decoration and aggregation. In stark contrast, EXF3 (Figure 2g,h), prepared in a lower concentration of cerium sulphate solution (0.1 M), displays a morphology dominated by clean, large graphene flakes with only sparse nanoparticle decoration.

As shown in the energy-dispersive spectra (Figure 2c,f,i) the presence of carbon, oxygen, and cerium confirms the developed nanocomposites’ structure. The elemental maps demonstrate a strong co-localization of cerium and oxygen signals, confirming the formation of cerium oxide nanoparticles distributed across the carbon scaffold. These results confirm that both electrolyte type and concentration have a strong influence on the final cerium loading during the in situ electrochemical exfoliation process of graphite.

In order to assess the crystalline characteristics of the graphene-based nanocomposites, X-ray diffraction analysis was carried out (please see Figure S1 in Electronic Supplementary Materials). The diffraction patterns show distinct variations across the examined samples, indicating that the synthesis parameters have a significant impact on the crystallinity and structural organization of the developed materials. In all samples the presence of graphene appears in the form of a relatively sharp diffraction peak centered at about 26.41°, corresponding to the (002) crystalline framework of multi-layer graphene structure. In case of EXF1 and EXF2 samples, beside graphene contribution, the diffractions exhibit more broad and low-intensity features, which can be attributed to the characteristic reflections of crystalline CeO_2_ nanoparticles. The coexistence of CeO_2_ reflections with the graphene-related peaks confirms the formation of a hybrid nanocomposite structure consisting of graphene sheets decorated with CeO_2_ nanoparticles. For EXF3 material the corresponding CeO_2_ diffraction lines are missing, indicating a higher level of structural ordering within the graphene framework and a lower loading of CeO_2_ nanoparticles, as already demonstrated through the microscopic analysis. Overall, the XRD results demonstrate that the synthesis conditions strongly influence both the crystallinity and distribution of CeO_2_ nanoparticles within the graphene matrix, leading to distinct structural characteristics across the prepared materials.

The chemical composition of cerium-containing graphene-based nanocomposites was evaluated through an XPS analysis, providing clear evidence for the existence of carbon, oxygen, and cerium atoms in the analyzed materials (please see the survey spectra depicted in Figure S2—Electronic Supplementary Materials). In all cases, the spectra are dominated by the characteristic signals corresponding to C 1s (~284–285 eV), O 1s (~530–532 eV), and Ce-related features, including the Ce 3d multiplet (~880–920 eV), as well as the Auger O KLL and Ce LMM signals. The presence of strong carbon signals confirms that the graphene scaffold is the primary structural component of all materials, whereas the oxygen signal reflects the contribution of cerium oxide nanoparticles as well as oxygen-containing functional groups associated with the partially oxidized graphene surface. Significant differences can be observed in the relative intensity of Ce-related features suggesting that the cerium loading varies in the analyzed hybrid materials. EXF1 has the most obvious Ce signals, which corresponds to the strongest cerium incorporation, whereas EXF3 has greatly attenuated Ce characteristics, indicating a lower nanoparticle decorating degree at the surface of graphene sheets. The elemental composition obtained from each survey spectra is summarized in Table S1 in Electronic Supplementary Materials, highlighting the strong dependence of surface composition on the synthesis conditions.

**Figure 3:**
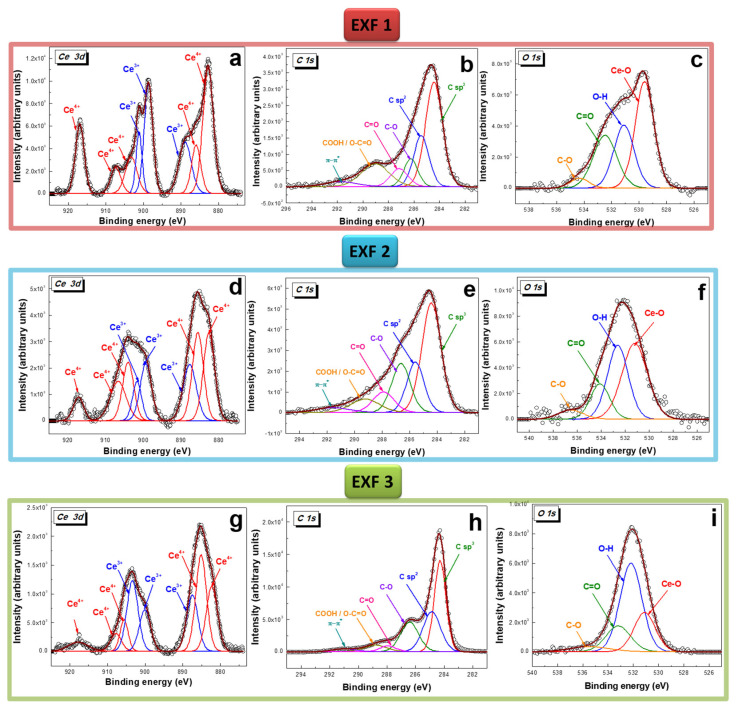
Deconvolution of high resolution Ce 3d, C 1s, and O 1s states of EXF1 (**a**–**c**); EXF2 (**d**–**f**); and EXF3 (**g**–**i**).

In order to differentiate between different chemical species, the deconvolution of C 1s, O 1s, and Ce 3d high resolution spectra was performed (please see Figure 3 and Table S2 in Electronic Supplementary Materials).

As visible in Figure 3a,d,g, the photoemission from the Ce 3d levels generates a relatively complex signal, embedding three major spectral features. For their deconvolution, previous reports were taken into account [45,46,47,48,49,50]. In all cases, well-separated spin–orbit components (with an energy splitting of about 18 eV) appear, accounting for the Ce 3d_3/2_ and Ce 3d_5/2_ states of CeO_2_. Further, each of these spin–orbit components is additionally split by multiplet splitting. According to the deconvolution, the recorded XPS signals consist of a combination of tri- and tetravalent oxidation states of Ce (Ce^3+^ and Ce^4+^). A clear proof for the presence of Ce^4+^ states is given by the existence of the high-binding energy peak located for all the analyzed samples, at about 917 eV, which strongly confirms the CeO_2_ structure of nanoparticles in the developed carbon-based nanostructures [51]. The assignment of the eight components and their characteristics can be found in Table S2 in Electronic Supplementary Materials.

The experimental C 1s spectrum (centered at about 284.78 eV for EXF1—Figure 3b; 284.64 eV for EXF2—Figure 3e; and 284.48 eV for EXF3—Figure 3h) was deconvoluted into six distinct components with a Gaussian–Lorentzian shape. In all cases, the spectra are dominated by a major *sp*^2^-hybridized carbon atom contribution, proving that most carbon atoms are in the conjugated honeycomb structure of graphene. In addition, structural disorder in the graphene structure appears in the form of carbon–hydrogen linked groups in a *sp*^3^-hybridized state. Furthermore, according to the chemical environment in which carbon atoms find themselves, carbon–oxygen-containing components were identified: C-O-C, C=O, O=C-O, and COOH [52,53] (please also see Table S2 in Electronic Supplementary Materials). The high-binding energy area showed a small contribution from the graphitic carbon π → π* shake-up satellite [54].

Four different peaks were necessary for the O 1s signals deconvolution. As visible in Figure 3c,f, for EXF1 and EXF2, the major contribution is given by the Ce-O states of CeO_2_ nanoparticles, confirming the morphology analysis results, which indicate a high cerium-containing nanoparticle content at the surface of these two nanocomposite materials. On the other hand, in the case of EXF3 (Figure 3i), the component attributed to Ce-O is considerably reduced, due to the lower content of nanoparticles present at the surface of the graphene sheets. In addition, other contributions can be assigned to hydroxyl (O-H), epoxy (C-O), and carboxylic (C=O) groups generated by the graphene lattice defects (please see Table S2 in Electronic Supplementary Materials) [55].

The C/Ce ratio was determined to be 0.0010 for EXF1, 0.0013 for EXF2, and 0.039 for EXF3, respectively, confirming that the highest cerium content was obtained in the case of the nanocomposite material prepared in exfoliation medium containing cerium nitrate hexahydrate (EXF1). The concentration of the exfoliation medium employed in the case of EXF2 and EXF3 induces major differences between the two samples. A lower concentration of the cerium sulphate electrolytic medium leads to a smaller amount of cerium-containing nanoparticles in the case of EXF3 compared to EXF2. Furthermore, the C/O ratio was calculated to be 0.22, 0.17, and 0.55 for EXF1, EXF2, and EXF3, respectively, reflecting differences in surface oxidation degree and oxygen-containing functional groups. The lower C/O ratios observed for EXF1 and EXF2 suggest a higher carbon lattice oxidation level, consistent with increased incorporation of oxygen from the cerium oxide nanoparticles and the employment of a more concentrated exfoliation media. Notably, the use of cerium sulphate as a precursor results in a more defective graphene structure compared to the material obtained in cerium nitrate solution. In contrast, the higher C/O ratio computed in case of EXF3 indicates a lower surface oxidation degree and a better preservation of the *sp*^2^-hybridized carbon structure since in this case the concentration of the exfoliation medium was lower.

To get a clearer picture of the surface redox states in the cerium-based nanoparticles, we examined the high-resolution Ce 3d spectra and added up the atomic concentrations associated with each oxidation state (Table S2 in Electronic Supplementary Materials). From this, we estimated the relative amounts of Ce^3+^ and Ce^4+^ in the three samples. EXF1 contained a little over 61% Ce^4+^ and about 39% Ce^3+^. EXF2 was the most oxidized of the group, with nearly 67% Ce^4+^. EXF3, on the other hand, had the highest share of Ce^3+^, approaching 44%. EXF1 exhibited 61.33% Ce^4+^ and 38.67% Ce^3+^, EXF2 showed the highest oxidation level, with 66.80% Ce^4+^ and 33.20% Ce^3+^, while EXF3 possessed the highest relative Ce^3+^ content, with 56.18% Ce^4+^ and 43.82% Ce^3+^.

To describe the nanomaterials in their biological testing environment, we performed DLS and Zeta potential analyses in culture medium supplemented with 5% FBS. The hydrodynamic diameters ranged between 890 and 2986 nm (please see Figure S3 in Electronic Supplementary Materials), significantly higher than the primary sizes observed in TEM, which is attributed to the formation of a protein corona and natural flake aggregation in high-salinity solutions. It is important to note that DLS overestimates the size of 2D materials like graphene, as the measurement assumes spherical particles, which also contributes to the observed high polydispersity index. Baseline measurements for free serum (15–25 nm) were within normal physiological ranges. For the culture medium (~315 nm) (Figure S4 in Electronic Supplementary Materials) the size was within normal physiological ranges, taking into consideration the presence of proteins, lipids, and microvesicles.

Zeta potential measurements confirmed the negative charge of the biological components. Bovine serum showed a moderately negative value (−13 mV) (Figure S5a from Electronic Supplementary Materials), which is characteristic of proteins like albumin. The culture medium exhibited a weaker negative potential (−8 mV) (Figure S5b in Electronic Supplementary Materials), because the high concentration of salts screens the surface charges. In the presence of graphene-containing materials (Figure S5c in Electronic Supplementary Materials), the potential decreased up to −23 mV, indicating a surface strongly negatively charged by graphene’s functional groups. This increased negative charge improves colloidal stability and explains the more negative values observed in the biological environment.

### 2.2. Biological Assay Investigation

In order to investigate whether these graphene-based nanomaterials are toxic in vitro, the cell viability, LDH activity, and ATP levels were measured, and oxidative stress parameters were quantified. The cell viability of normal dermal fibroblasts (BJ) and human hepatocarcinoma cells (HepG2) was assessed in response to varying concentrations of the cerium-containing graphene-based nanomaterials prepared employing different electrolyte exfoliation medium at varying concentrations (EXF1 in a 0.2 M Ce(NO_3_)_3_ solution, EXF2 in a 0.2 M Ce(SO_4_)_2_ solution, and EXF3 in a 0.1 M Ce(SO_4_)_2_ solution) in order to determine the potential influence of the exfoliation conditions on the biological response.

**Figure 4:**
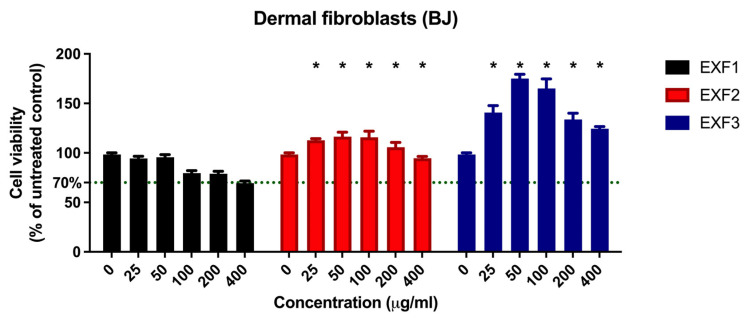
Viability of dermal fibroblasts exposed to cerium-containing graphenes denoted EXF1, EXF2, and EXF3 in concentrations ranging between 25 and 400 µg/mL. Data is presented as % of untreated controls (n = 3 ± SD). The horizontal dashed line at 70% represents the cytotoxicity threshold. Asterisks above data points indicate significant differences from the untreated control (*p* < 0.05).

When BJ fibroblasts were exposed to the cerium-containing nanocomposites, viability results were improved compared to the untreated controls revealing a concentration-dependent response (Figure 4). In all experimental groups, the IC_50_ (half-maximal inhibitory concentration) was not reached within the tested range of 0–400 µg/mL, indicating a low toxicity profile. The established cytotoxicity threshold was 70% viability (representing 30% inhibition of cell growth) and the IC_30_ was instead calculated (Table 1). EXF1 remained largely inert; since it maintained cell viability close to or slightly below 100% up to 100 µg/mL, followed by a moderate decrease at higher concentrations, with values approaching the 70% cytotoxicity threshold at 400 µg/mL (Figure 4). Notably, EXF2 and EXF3 induced a significant hormetic effect in fibroblasts [56] (Figure 4), highlighting the selectivity observed when comparing their effects to the inhibitory response in HepG2 cells (Table 1). The viability of the treated fibroblasts was increased significantly above the control level for concentrations up to 200 µg/mL (EXF2) and 400 µg/mL (EXF3). As visible in Figure 4, EXF3, obtained using the lower cerium sulfate concentration (0.1 M), exhibited a distinctive hormetic-like response, with a maximal stimulation exhibiting a strong proliferative response obtained in cells exposed at intermediate concentrations (50 µg/mL). Two-way ANOVA evaluation showed significant material and concentration-induced effects (*p* < 0.0001).

**Figure 5:**
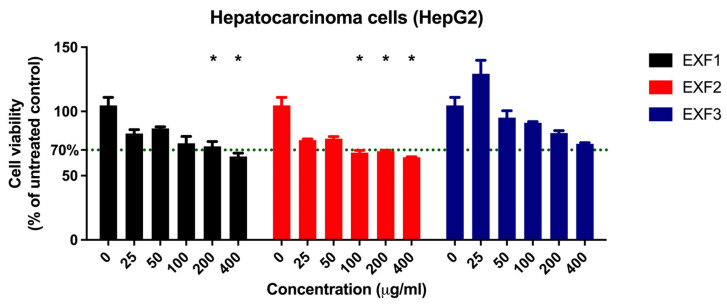
Viability of hepatocarcinoma cells exposed to cerium-containing graphenes, denoted EXF1, EXF2, and EXF3 in concentrations ranging between 25 and 400 µg/mL. Data is presented as % of untreated controls (n = 3 ± SD). The horizontal dashed line at 70% represents the cytotoxicity threshold. Asterisks above data points indicate significant differences from the untreated control (*p* < 0.05).

For HepG2 hepatocarcinoma cells, two-way ANOVA showed that the investigated materials exhibited a concentration-dependent cytotoxic response (*p* < 0.0001) (Figure 5). The strongest inhibitory effect was observed for EXF1 and EXF2, having a higher ceria content, as cell viability decreased across all tested concentrations. Higher concentrations (above 100 µg/mL) induced significant toxic effects, crossing the 70% viability threshold (Figure 5). In contrast, EXF3 displayed a milder effect, even exhibiting a minimal cytostimulation on HepG2-treated cells at 25 µg/mL, while still inducing cytotoxicity at higher doses (Figure 5). However, it remained above the toxicity limit across the tested range.

**Figure 6:**
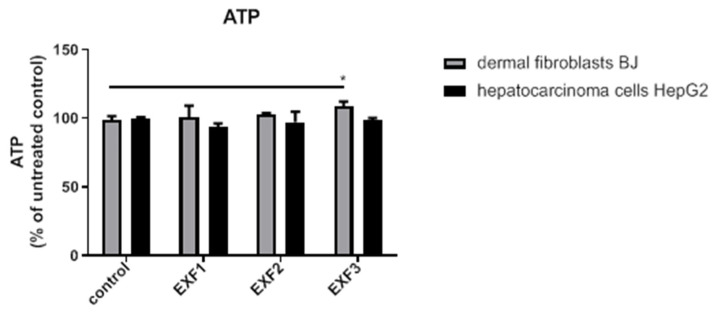
Comparative assessment of the ATP levels for BJ (dermal fibroblasts) and HepG2 (hepatocarcinoma cells) and control cells after exposure to the same concentration of 50 µg/mL graphene-based materials. Data are presented as mean ± SD (n = 3); asterisks above data points indicate significant differences from the untreated control (*p* < 0.05).

Cell membrane integrity was evaluated by LDH activity measurements [55] (Table 2). There were no significant differences between the control and dose of 50 µg/mL EXF1, EXF2, and EXF3. Lower LDH values were seen when BJ cells were incubated with all graphene-based materials while in Hep2 cells LDH activity increased, but it was statistically insignificant in all groups. These results are concordant with those obtained in viability assay and suggest that cerium-containing graphene-based nanomaterials did not induce membrane lesions. The measurement of adenosine triphosphate (ATP) levels (Figure 6), a quantitative marker of cellular metabolism, further supports the observed divergent viability patterns. In BJ fibroblasts, there was a significant increase in ATP production for the EXF3 group (108.8 ± 3.5 U/I; *p* < 0.05), which is concordant with the observed cytostimulatory effect. Conversely, EXF1 induced significantly decreased levels of ATP in the hepatocarcinoma line (93.8 ± 2.4 U/I; *p* < 0.05), correlating with the inhibitory effects exerted in these cells.

The redox status in cells was evaluated through the measurement of malondialdehyde (MDA) levels and quantification of antioxidant enzyme activity (CAT and SOD) after treatment with the three graphenes. Our results indicate that the investigated nanomaterials exert a relatively uniform pro-oxidative stimulus, particularly for the EXF2 and EXF3 formulations, where significant increases in lipid peroxidation (MDA) were observed in both cell lines, but the adaptative antioxidant response varied. In BJ cells, MDA, an end product of lipoperoxidation, increased after EXF2 incubation in parallel with significant improvement of CAT activity (*p* < 0.05), which likely contributed to maintaining increased cell viability despite redox imbalance. In HepG2 cells, both EXF2 and EXF3 were associated with lipid peroxidation (*p* < 0.05) and adaptive SOD activity enhancement, especially after EXF3 incubation, suggesting that high values of cellular viability may correlate with effective intracellular antioxidant defense. These data suggest that oxidative stress can be an important mechanism of toxicity triggered by graphenes, but the minimal consequences in cell viability might be explained by the cellular antioxidant response (Table 3).

To validate the redox-modulatory effects of the nanocomposites, an in vitro hydrogen donor assay was performed on cell lysates [57]. In normal BJ fibroblasts, EXF1 and EXF2 treatment resulted in an increase in radical scavenging activity (35.13% and 33.72%, respectively) compared to the control (29.73%), whereas EXF3 induced a slight reduction to 26.73%. Conversely, in HepG2 hepatocarcinoma cells, all three materials led to a depletion of the antioxidant defenses. Relative to the HepG2 control baseline of 38.43%, radical inhibition decreased to 30.71% for EXF1 and 23.74% for EXF2, with the most pronounced depletion observed in EXF3-treated cells, where the inhibition percentage dropped to 19.18%.

## 3. Discussion

The primary objective of this study was to evaluate the biological effects of three distinct cerium-containing graphene nanocomposites obtained via liquid-phase graphite electrochemical exfoliation. The morphological and surface chemical analyses revealed that the exfoliation strategy in cerium salts electrolyte media enabled the direct formation of cerium-decorated graphene sheets. Depending on the precursor type and its concentration, the obtained materials revealed significant characteristic differences. TEM, SEM, and STEM-EDX analyses confirmed the presence of thin, wrinkled graphene sheets decorated with cerium-containing nanoparticles whose density and distribution strongly depended on the electrolyte type and concentration. The sample obtained in a 0.2 M cerium nitrate medium (EXF1), exhibited a highly aggregated nanoparticle coverage, while EXF2, synthesized in a 0.2 M cerium sulphate solution, displayed a moderately decorated graphene surface. Conversely, EXF3, synthesized at a lower cerium sulphate concentration (0.1 M), showed large, clean graphene flakes sparsely decorated with isolated cerium oxide nanoclusters. These findings clearly indicate that the electrochemical exfoliation environment governs both the cerium incorporation degree and the resulting nanostructure morphology. In this context, the aim of the current study was to demonstrate that by fine-tuning the synthesis parameters, we can control the obtained nanocomposite physicochemical properties and subsequently modulate its biological responses.

Compared to prior studies assessing cerium-functionalized graphenes’ toxicity where the materials were obtained via traditional ex situ methods, which involve multiple synthesis steps since the cerium doping is obtained through decoration of pre-existing graphene oxide (GO) or reduced graphene oxide (rGO), the in situ electrochemical exfoliation method reported here offers potential advantages [8,42,43,44]. This one-step approach is an environmentally friendly, green process [58,59], since the reaction takes place at room temperature, with minimum chemical precursors and energy consumption, with graphite exfoliation and the doping of the graphene sheets occurring simultaneously [60]. The resulting nanocomposite material presents less contamination that is often introduced during transfer processes in the classical methods [61,62]. Because the synthesis parameters, including applied potential, electrolyte composition, and concentration, can be precisely adjusted to customize the exfoliation degree, surface chemistry, and dopant incorporation, this method also offers improved control over the final nanocomposite’s physicochemical characteristics [59,61,62].

The distinct physicochemical properties and morphology of our as-prepared materials determined diverse biological outcomes in normal dermal fibroblasts, BJ, and human hepatocarcinoma cells, HepG2. The cellular responses observed in this study provide preliminary insights into the differential activity of these hybrids. Our results, although providing moderate and formulation-dependent biological responses, do align with prior studies, which revealed favorable profiles of cerium–graphene hybrids, including high biocompatibility and selective cytotoxic activity in multiple cancer-cell lines [8,42,43,44]. A unique finding in our study, however, was a cyto-proliferative response in fibroblast cells after exposure to two of the compounds (EXF2 and EXF3) in correlation with increased ATP levels and CAT activity, especially in EXF3-treated BJ cells.

The synthesis parameters (the choice of electrolytic medium—nitrate versus sulfate—and its concentration) directly determined the composition and morphology of each nanocomposite. High electrolyte concentrations (0.2 M) used for synthesizing EXF1 and EXF2 resulted in high cerium loading, while lower concentration resulted in significantly decreased cerium loading obtained in the case of EXF3. Furthermore, XPS analysis revealed differences in the surface redox state (Ce^3+^/Ce^4+^ ratios) among the synthetized formulations. Since the redox reactivity of cerium oxide materials is influenced by the Ce^3+^/Ce^4+^ balance [63,64], these changes in oxidation state are significant, potentially influencing the antioxidant or pro-oxidant capacity [37]. A Ce^4+^-rich surface may favor pro-oxidant activity by increasing ROS formation, while a higher content of Ce^3+^ might mimic antioxidant behavior through effective ROS scavenging [65]. Thus, our findings suggest that surface redox composition and local oxidative environment may contribute to the distinct biological responses observed across the reported compounds. While the changes in MDA and antioxidant enzyme activity point toward a redox-mediated pathway, further studies utilizing direct ROS quantification or mitochondrial membrane potential analysis are required to fully explain the biological effects.

The overall biological profiles of the compounds (EXF1, EXF2, EXF3) differed. Both EXF1 and EXF2 presented selective cytotoxicity in hepatocarcinoma cells, but EXF1 remained largely inert when exposed to fibroblasts, while EXF2 induced a significant hormetic, potentially due to the adaptive increase in antioxidant defense in cells. This difference might also be linked to the relative abundance of Ce^3+^ and Ce^4+^ states in the two materials, which is determined by different synthesis conditions. A biphasic hormetic effect, characterized by an initial increase in cellular survival at lower doses (25–50 μg/mL) as an adaptive response to mild stress, followed by a decrease in survival when the dose of nanomaterials exceeded the cell’s compensatory mechanisms, was observed in both the high-loading EXF2 and the low-loading EXF3 (Figure 4). This effect was significantly more sustained in EXF3 and correlated with increased ATP levels and CAT activity in cells. The magnitude of this hermetic-like cytostimulatory effect appeared inversely correlated with cerium loading, with EXF3 having the most pronounced effect. EXF3 was characterized by lower cerium content, sparser nanoparticle decoration, and the highest relative Ce^3+^ content among the investigated compounds.

We demonstrated the efficiency of one-step electrochemical exfoliation approach in synthetizing cerium-containing graphene-based nanocomposites. The physicochemical characteristics of the materials were tuned by adjusting the synthesis parameters, including the precursor salt, concentration, and applied voltage. We used three distinct nanocomposite formulations that presented significant variations in their properties, including morphology, cerium nanoparticles density, and surface redox states (Ce^3+^/Ce^4+^ ratio), allowing for the establishment of a preliminary structure–biomedical activity relationship. We acknowledge that a wider array of compounds would provide more mechanistic insights, but the aim of this study was to demonstrate that biological activity of these hybrids can be actively tuned.

Therefore, our findings extend beyond the prevailing narrative, which has mainly focused on achieving graphene ‘biocompatibility’ or ‘neutrality’, allowing for application-specific material design. Furthermore, this environmentally friendly preparation protocol allows for functionalization under mild conditions, thus overcoming inherent cytotoxicity, one of the main obstacles to graphene’s clinical application. Previous reports showed that functionalization with cerium nanoparticles mitigates the inherent toxicity of graphene scaffold while also offering selective anticancer activity [42,43,44]. However, our approach offers a straightforward strategy for engineering nanocomposites’ redox-responsiveness, going beyond the conventional objective of only obtaining graphene biocompatibility. The material’s biological effect can be actively modulated by directly controlling the synthesis parameters, optimizing functional roles for a multitude of purposes, as biological effects—such as cytotoxicity versus cytostimulation—are primarily dependent on the material’s physicochemical properties. The divergent biological profiles observed for these formulations provide a physicochemical rationale for evaluating such hybrids within established pre-clinical domains, suggesting that these nanocomposites can be leveraged for multiple applications once fully validated and understood mechanistically.

In regenerative medicine, where both CeO_2_-NPs and graphene have previously shown promise in wound healing and tissue engineering, these hybrid compounds can be tuned for an additional stimulatory effect, as seen with EXF2 and EXF3 [5,19,66,67,68,69,70]. In oncological research, graphene materials are already used as high-capacity vehicles for targeted and controlled drug delivery [5,9,71]. Our findings suggest that these platforms could potentially be investigated for further optimization and theireffects could be leveraged to potentially enhance the efficacy of co-administered drugs as part of a multifunctional system, rather than serving as a standalone potent chemotherapeutic agent. Moreover, as both components (CeO_2_ and graphene) possess optical, redox, and electronic properties, the reported nanocomposites may find applicability in bioimaging or biosensing [72,73,74].

In summary, the synthesis of cerium–graphene hybrids via electrochemical exfoliation allows for the development of tunable compounds that can be tailored for specific biomedical applications. While our preliminary results are intriguing, further research is required to provide mechanistic insights into the hormesis and selective toxicity following exposure to such compounds, as these modifications appear to be cell-specific.

Despite the distinct biological patterns observed, a limitation of the current study is the lack of direct evidence regarding cellular internalization and intracellular localization. Our results suggest that membrane integrity remained largely intact, as evidenced by LDH activity. However, we did not directly quantify the uptake efficiency or identify the specific intracellular compartments where these nanocomposites accumulate and therefore, the specific role of internalization in our proposed structure-activity relationship remains to be fully elucidated. We acknowledge this as a preliminary stage of research, and future studies should employ methods to correlate the materials’ physicochemical properties with their intracellular fate.

## 4. Materials and Methods

### 4.1. Reagents

Cerium(III) nitrate hexahydrate (Ce(NO_3_)_3_·6H_2_O, 99.999% trace metal basis), cerium(IV) sulfate (Ce(SO_4_)_2_, 99.9%), and methanol HPLC grade (CH_3_OH) were purchased from Alfa-Aesar (Kandel, Germany) while graphite rods (6 mm diameter, 99.995% purity), sodium nitrate (NaNO_3_, ≥99.9%), and ammonium sulfate ((NH_4_)_2_SO_4_, ≥99.0%) were obtained from Sigma-Aldrich (Taufkirchen, Germany). Ultrapure water (18.2 MΩ) was provided by a Milli-Q water purification system (Millipore Corp., Bedford, MA, USA). Spectra/Por^®^ 6 MWCO 1000, 45 mm dialysis membrane was acquired from Carl Roth (Karlsruhe, Germany). Malondialdehyde colorimetric assay kit, catalase activity kit, and superoxide dismutase activity kit were purchased from Elab Science, Wuhan, China.

### 4.2. Synthesis of Cerium-Doped Graphene-Based Nanomaterials

For the production of three different cerium-containing graphene-based materials, simple, quick, economically advantageous, and environmentally friendly protocols were established based on liquid-phase electrochemical exfoliation of high purity graphite rods in electrolyte solutions containing rare earth salts. For the first material production, henceforth denoted EXF1, the exfoliation process was performed in the presence of a cerium nitrate hexahydrate solution. The electrolyte was prepared by dissolving 8.684 g of cerium nitrate hexahydrate powder (Ce(NO_3_)_3_·6H_2_O) in 100 mL ultrapure water under magnetic stirring at room temperature to obtain a homogeneous salt aqueous solution at a concentration of 0.2 M. The other two graphene-based materials (further denoted EXF2 and EXF3) were obtained employing a cerium (IV) sulfate (Ce(SO_4_)_2_) solution at two different concentrations: 0.2 M and 0.1 M, respectively. In this case, the electrolytes were prepared at room temperature by dissolving 6.644 g (for 0.2 M) or 3.322 g (for 0.1 M), respectively, of (Ce(SO_4_)_2_) in 100 mL ultrapure water under continuous magnetic stirring. In all performed exfoliation experiments, a 150 mL glass beaker with a homemade Teflon cover (provided with two holes centered at a distance of 2 cm) was used to build the electrochemical cell. Two pure graphite rods were immersed in the electrolyte, fixed in the Teflon cover, and served as anode and cathode. A low bias was applied to the graphite rods. For the first exfoliation experiment, the applied voltage was 5 V, which generated a current of ≈0.5 A. Initially, the electrolyte was colorless. Shortly after applying the voltage, it became yellowish, and the first exfoliated flakes appeared 30 min after the experiment started. Then, the quantity of the exfoliated material increased, and the solution turned black. The experiment was stopped after a total of 5 h; the graphite rods were removed, and the obtained solution was allowed to set overnight at room temperature. The next day, the exfoliated material had settled at the bottom of the beaker, the supernatant was removed, and the material was washed with 5 L of ultrapure water. In the last washing step, the total volume of the solution was reduced to 50 mL, and the colloidal suspension was transferred to a dialysis membrane (SPECTRA/POR 6MWCO) and immersed in ultrapure water. The water was periodically replaced for the next 3 days, until a neutral pH was reached. In the end, the solution was removed from the membrane, frozen, and dried through lyophilization (Christ Alpha 1-4 LSC Freeze Dryer—Martin Christ Gefriertrocknungsanlagen GmbH, Osterode am Harz, Germany) resulting in a black powder—EXF1. For obtaining EXF2 and EXF3, the applied voltage was 6 V, generating a current of 0.7 A when the electrolyte solution was 0.2 M (Ce(SO_4_)_2_) and 0.4 A when the concentration was lower (0.1 M Ce(SO_4_)_2_). The same procedure employed for EXF1 was followed for obtaining the final black powder materials.

### 4.3. Instruments

A SU-8230 STEM system (Hitachi High-Technologies Corp., Tokyo, Japan) was used to conduct transmission electron microscopy (TEM), scanning electron microscopy (SEM), and scanning transmission electron microscopy (STEM) with energy-dispersive X-ray spectroscopy (EDX) for the elemental mapping of C, O, and Ce atom distribution. The X-ray diffraction (XRD) measurements were carried out using a Rigaku-SmartLab automated multipurpose X-ray diffractometer (Rigaku Corporation, Tokyo, Japan) with Cu-Kα radiation, operating at 45 kV, 200 mA. The processing of diffraction patterns was performed using PDXL2: Integrated X-ray powder diffraction software. A SPECS spectrometer (SPECS Surface Nano Analysis, GmbH, Berlin, Germany) equipped with a dual-anode X-ray source Al/Mg, a PHOIBOS 150 2DCCD hemispherical energy analyzer, and a multi-channeltron detector was employed to determine the elemental composition of the cerium-containing graphene-based nanocomposites.

The samples, as a colloidal suspension in methanol (HPLC grade), were dried in consecutive layers on indium foil previously mounted to wolfram sample holder with carbon tape. X-ray photoelectron spectroscopy (XPS) measurements were taken under AlKα X-ray source (1486.6 eV) irradiation, at 200 W. During measurement the sample was stored inside the measurement chamber at a constant pressure of roughly 1 × 10^−9^ torr. Surface cleaning was achieved through bombarding with argon ions at 500 V for 5 min. As the samples exhibited negligible electrostatic charge, the binding energies are provided as collected. Furthermore, the raw data were used without any preliminary smoothing. The Ce 3d, C 1s, and O 1s spectra were analyzed employing Casa XPS software, version 2.3.16 (Casa Software Ltd., Wilmslow, Cheshire, UK), taking into account a non-linear Shirley background correction and a Gaussian–Lorentzian product function for the components.

### 4.4. Biological Assay

The cytotoxic effect of cerium-containing graphene-based hybrid materials was evaluated in vitro on two types of cells, normal dermal fibroblasts (BJ-CRL- 2522- ATCC, Gaithersburg, MA, USA) and human hepatocarcinoma cells, HepG2 (HepG2-HB-8065, ATCC).

#### 4.4.1. Cell Culture

The normal dermal fibroblasts (BJ) and human hepatocarcinoma (HepG2) cell lines were cultivated in DMEM (Dulbecco’s modified Eagle medium with high glucose) supplemented with 5% FCS, streptomycin, penicillin, and amphotericin under standard culture conditions. Medium was changed twice a week. All reagents were bought from Biochrom AG, Berlin, Germany. HepG2 cells are frequently used as an in vitro model of human hepatocytes for toxicity assays [75].

#### 4.4.2. Cell Viability

Cells (BJ and HepG2) were cultivated in 96-well plates at a density of 5 × 10^4^ cells/well, allowed to acclimate for 24 h under standard conditions, then exposed to different concentrations of each nanohybrid material suspended in fresh medium. The samples were subjected to sonication before the preparation of the final solutions for cell exposure to ensure a homogeneous dispersion. The solutions were made immediately before the exposure. After the exposure to the materials for 24 h, cells were washed with PBS (phosphate-buffered saline), then fresh medium was added, and toxicity was assessed by using the neutral red toxicology assay kit (TOX4 1KT, Sigma) as indicated by the manufacturer. The assay allows for the estimation of the viable cells, which incorporate the dye, as opposed to non-viable cells, and it is included in the 3T3-NRU-phototoxicity-assay (OECD no. 432) guideline test [75,76]. Absorbance was read at 540 nm by using the SpectraMax iD3 Multi-Mode Microplate Reader IDmax plate reader (Molecular Devices, Salzburg, Austria). The experiments were done in triplicate. All biological assays were performed in triplicate (technical triplicates) to ensure experimental precision. The results are presented as % of the untreated control; a dose that caused a viability decrease below 70% was considered toxic.

#### 4.4.3. LDH and ATP Assessment

Lactate dehydrogenase (LDH) is a marker of cell membrane damage marker and its activity was evaluated from the cell culture medium using the spectrophotometric method [57]. Absorbance was measured at 340 nm. LDH activity was expressed in units (the amount required to catalyze the reduction of 1 μmol NAD/min·mg protein was considered one unit of activity). ATP levels were measured using CellTiter-Glo™ Luminescent Cell Viability Assay Kit (Promega, Madison, WI, USA). At the end of graphene exposure, cells were processed according to the manufacturer instructions. ATP readings were done in luminescence by using the Spectra Max ID3 ELISA plate reader (Molecular Devices LLC, San Jose, CA, USA). The results are expressed as % of the control, experiments were performed in triplicate.

#### 4.4.4. Cell Lysates

Cells exposed for 24 h to 50 µg/mL of cerium-containing graphene-based nanomaterials were collected by scraping on ice, further washed, and exposed to a lyses buffer containing Igepal-Nonidet 1% (Sigma), 1% protease inhibitor complex (Sigma) in phosphate-buffered saline (PBS). Supernatant was collected through centrifugation. Protein content was determined through the Bradford method (Bio-Rad Laboratories, Inc., Hercules, CA, USA). All experiments were performed in triplicate.

#### 4.4.5. Oxidative Stress Assessment

Malondialdehyde (MDA) was measured by using the Malondialdehyde (MDA) Colorimetric Assay Kit (TBA Method) (Elab Science, Wuhan, China). The measurement of the enzymatic activities of the superoxide dismutase (SOD) was done from cell lysates by using the Total Superoxide Dismutase (T-SOD) Activity Assay Kit from (Elab Science) and respectively catalase (CAT) using the Catalase (CAT) Activity Assay Kit (Elab Science).

Readings were done at 450 nm, correction wavelength 540 nm, using Spectra Max ID3 ELISA plate reader; the results are presented as MDA—nMoles/mg protein, SOD, and CAT—units/mg protein.

### 4.5. Statistical Analysis

All statistical analyses and graph generation were conducted under GraphPad Prism Version 10.3.1 for Windows (GraphPad Software, San Diego, CA, USA). Prior to statistical testing, data normality and homogeneity of variance were verified using the Shapiro-Wilk test. The results are presented as mean ± standard deviation (SD). The data were analyzed using two-way ANOVA followed by Bonferroni’s post hoc test for multiple comparisons between groups and concentrations. A *p*-value < 0.05 was considered statistically significant.

## 5. Conclusions

This study establishes in situ electrochemical exfoliation as a versatile preparation protocol for synthesizing cerium-containing graphene-based nanocomposites with distinct physicochemical properties. Herein, we report the synthesis of three different materials. Morphological and structural investigations revealed that the electrolyte content and composition play a significant role in determining the resultant material’s surface oxidation state (Ce^3+^/Ce^4+^ ratio), dopant dispersion and loading, these factors further defining the biological responses observed in normal (BJ) and malignant (HepG2) cell lines.

We demonstrated that the investigated materials determine distinct and cell-specific responses, ranging from selective anticancer cytotoxicity to pronounced increase in viability in normal fibroblasts. Highly oxidized, Ce^4+^-rich formulations demonstrated increased cytotoxicity towards cancer cell lines. This preliminary finding suggests that the redox state potentially plays a role in regulating biological outcomes. Furthermore, materials with a higher Ce^3+^ surface content and a lower nanoparticle density demonstrated a hormetic-like, proliferative effect in fibroblasts, possibly due to increased ATP levels and high antioxidant effect of CAT in cytosol. The observed effects are more compatible with surface-mediated redox interactions, even if a limited degree of ion release in biological media cannot be completely ruled out despite the fact that cerium is mostly present as surface-bound CeO_2_ species.

As an exploratory investigation, this work serves as a foundational proof of concept, demonstrating that the biological activity of graphene-based hybrids can be tuned. By achieving distinct biological outcomes through subtle modifications in the synthesis process, this study highlights the importance of comprehensive material characterization in predicting biological effects. Therefore, such nanocomposites show promising potential for further use in specific biomedical purposes such as regenerative medicine, where regulated hormetic stimulation may improve wound healing and tissue repair, as well as oncology, where selective cytotoxicity can be used in anticancer treatment and drug delivery. This strategy unlocks the potential for translating graphene into clinical practice by mitigating its intrinsic toxicity through functionalization.

## Figures and Tables

**Table 1:** Calculated IC_30_ values (μg/mL for EXF1, EXF2, and EXF3 compounds in normal BJ fibroblasts and hepatocarcinoma (HepG2) cell lines). The IC_30_ represents the concentration resulting in 70% cell viability; IC_50_ values were not reached within the tested range (>400 μg/mL).

Compound	BJ Fibroblasts	HepG2
EXF1	386.0 µg/mL	246.4 µg/mL
EXF2	>400 µg/mL	130.9 µg/mL
EXF3	>400 µg/mL	>400 µg/mL

**Table 2:** LDH activity of BJ (dermal fibroblasts) and HepG2 (hepatocarcinoma cells) after exposure to graphene-based materials (50 µg/mL). Data is presented as mean ± SD (n = 3); statistical analysis (two-way ANOVA) showed no significant differences between the treated groups and the control (*p* > 0.05).

Treatment	BJ	HepG2
Control	119.3 ± 4.6	150.1 ± 6.7
EXF1	128.7 ± 3.7	155.1 ± 6.5
EXF2	128.2 ± 3.9	148.9 ± 4.0
EXF3	123.4 ± 2.1	162.9 ± 5.9

**Table 3:** Oxidative stress parameters (MDA, CAT, and SOD) of BJ (dermal fibroblasts) and HepG2 (hepatocarcinoma cells) after exposure to graphene-based materials (50 µg/mL). Data are presented as mean ± SD (n = 3); asterisks indicate significant differences from the untreated control (*p* < 0.05).

Cell Line	Treatment	MDA (nmol/mg Protein)	CAT (U/mg Protein)	SOD (U/mg Protein)
**BJ**	Control	0.375 ± 0.011	23.68 ± 1.00	42.41 ± 1.05
	EXF1	0.333 ± 0.012 *	24.68 ± 1.02	39.31 ± 0.64 *
	EXF2	0.425 ± 0.012 *	29.53 ± 0.80 *	35.63 ± 0.62 *
	EXF3	0.393 ± 0.009	21.60 ± 1.12	44.39 ± 1.05
**HepG2**	Control	0.487 ± 0.012	18.65 ± 0.88	38.82 ± 0.55 *
	EXF1	0.463 ± 0.009	23.78 ± 0.84 *	44.53 ± 1.23 *
	EXF2	0.573 ± 0.013 *	26.92 ± 0.99 *	38.44 ± 0.86
	EXF3	0.596 ± 0.006 *	25.89 ± 1.34 *	56.69 ± 0.98 *

## Data Availability

The datasets analyzed in the current study are available from the corresponding author on request.

## Supplementary Materials

The following supporting information can be downloaded at https://www.mdpi.com/article/10.3390/ijms27114772/s1.

## Abbreviations

The following abbreviations are used in this manuscript:

CAT	catalase
CeO_2_	cerium oxide (Nanoceria)
EDX	energy-dispersive X-ray spectroscopy
GBNs	graphene-based nanomaterials
GFNs	graphene-family nanomaterials
GO	graphene oxide
GQDs	graphene quantum dots
LDH	lactate dehydrogenase
MAPK	mitogen-activated protein kinase
MDA	malondialdehyde
nGO	nano-graphene oxide
NIR	near-infrared
rGO	reduced graphene oxide
ROS	reactive oxygen species
SOD	superoxide dismutase
STEM	scanning transmission electron microscopy
STEM-EDX	scanning transmission electron microscopy with energy-dispersive X-ray spectroscopy
TEM	transmission electron microscopy
XPS	X-ray photoelectron spectroscopy

## References

[1] Novoselov K.S., Geim A.K., Morozov S.V., Jiang D., Zhang Y., Dubonos S.V., Grigorieva I.V., Firsov A.A. (2004). Electric field effect in atomically thin carbon films. Science.

[2] Stergiou A., Cantón-Vitoria R., Psarrou M.N., Economopoulos S.P., Tagmatarchis N. (2020). Functionalized graphene and targeted applications—Highlighting the road from chemistry to applications. Prog. Mater. Sci..

[3] Han S., Sun J., He S., Tang M., Chai R. (2019). The application of graphene-based biomaterials in biomedicine. Am. J. Transl. Res..

[4] Kumar R., Singh D.P., Muñoz R., Amami M., Singh R.K., Singh S., Kumar V. (2023). Graphene-based materials for biotechnological and biomedical applications: Drug delivery, bioimaging and biosensing. Mater. Today Chem..

[5] Zhao H., Ding R., Zhao X., Li Y., Qu L., Pei H., Yildirimer L., Wu Z., Zhang W. (2017). Graphene-based nanomaterials for drug and/or gene delivery, bioimaging, and tissue engineering. Drug Discov. Today.

[6] Ou L., Song B., Liang H., Liu J., Feng X., Deng B., Sun T., Shao L. (2016). Toxicity of graphene-family nanoparticles: A general review of the origins and mechanisms. Part. Fibre Toxicol..

[7] Nemati F., Rezaie M., Tabesh H., Eid K., Xu G., Ganjali M.R., Hosseini M., Karaman C., Erk N., Show P.L. (2022). Cerium functionalized graphene nano-structures and their applications; A review. Environ. Res..

[8] Saranya J., Saminathan P., Ankireddy S.R., Shaik M.R., Khan M., Khan M., Shaik B. (2023). Cerium oxide/graphene oxide hybrid: Synthesis, characterization, and evaluation of anticancer activity in a breast cancer cell line (MCF-7). Biomedicines.

[9] Xu Z., Wang S., Li Y., Wang M., Shi P., Huang X. (2014). Covalent functionalization of graphene oxide with biocompatible poly(ethylene glycol) for delivery of paclitaxel. ACS Appl. Mater. Interfaces.

[10] Goenka S., Sant V., Sant S. (2014). Graphene-based nanomaterials for drug delivery and tissue engineering. J. Control. Release.

[11] Feng L., Zhang S., Liu Z. (2011). Graphene based gene transfection. Nanoscale.

[12] Saharan R., Paliwal S.K., Tiwari A., Tiwari V., Singh R., Beniwal S.K., Dahiya P., Sagadevan S. (2023). Exploring graphene and its potential in delivery of drugs and biomolecules. J. Drug Deliv. Sci. Technol..

[13] Khakpour E., Salehi S., Naghib S.M., Ghorbanzadeh S., Zhang W. (2023). Graphene-based nanomaterials for stimuli-sensitive controlled delivery of therapeutic molecules. Front. Bioeng. Biotechnol..

[14] Lee S.Y., Kwon M., Raja I.S., Molkenova A., Han D.W., Kim K.S. (2022). Graphene-based nanomaterials for biomedical imaging. Adv. Exp. Med. Biol..

[15] Lin J., Chen X., Huang P. (2016). Graphene-based nanomaterials for bioimaging. Adv. Drug Deliv. Rev..

[16] Yang K., Feng L., Shi X., Liu Z. (2013). Nano-graphene in biomedicine: Theranostic applications. Chem. Soc. Rev..

[17] Wang H., Xie G., Ying Z., Tong Y., Zeng Y. (2015). Enhanced mechanical properties of multi-layer graphene filled poly(vinyl chloride) composite films. J. Mater. Sci. Technol..

[18] Chen X., Zou M., Liu S., Cheng W., Guo W., Feng X. (2024). Applications of Graphene Family Nanomaterials in Regenerative Medicine: Recent Advances, Challenges, and Future Perspectives. Int. J. Nanomed..

[19] Kenry A., Lee W.C., Loh K.P., Lim C.T. (2018). When stem cells meet graphene: Opportunities and challenges in regenerative medicine. Biomaterials.

[20] Li Y., Yuan H., von dem Bussche A., Creighton M., Hurt R.H., Kane A.B., Gao H. (2013). Graphene microsheets enter cells through spontaneous membrane penetration at edge asperities and corner sites. Proc. Natl. Acad. Sci. USA.

[21] Sydlik S.A., Jhunjhunwala S., Webber M.J., Anderson D.G., Langer R. (2015). In vivo compatibility of graphene oxide with differing oxidation states. ACS Nano.

[22] Li Y., Liu Y., Fu Y., Wei T., Le Guyader L., Gao G., Liu R.S., Chang Y.Z., Chen C. (2012). The triggering of apoptosis in macrophages by pristine graphene through the MAPK and TGF-beta signaling pathways. Biomaterials.

[23] Vallabani N.V., Mittal S., Shukla R.K., Pandey A.K., Dhakate S.R., Pasricha R., Dhawan A. (2011). Toxicity of graphene in normal human lung cells (BEAS-2B). J. Biomed. Nanotechnol..

[24] Mendonça M.C., Soares E.S., de Jesus M.B., Ceragioli H.J., Ferreira M.S., Catharino R.R., da Cruz-Höfling M.A. (2015). Reduced graphene oxide induces transient blood-brain barrier opening: An in vivo study. J. Nanobiotechnol..

[25] Mao L., Hu M., Pan B., Xie Y., Petersen E.J. (2016). Biodistribution and toxicity of radio-labeled few layer graphene in mice after intratracheal instillation. Part. Fibre Toxicol..

[26] Liu J.H., Yang S.T., Wang H., Chang Y., Cao A., Liu Y. (2012). Effect of size and dose on the biodistribution of graphene oxide in mice. Nanomedicine.

[27] Zhang X., Yin C., Peng C., Hu W., Zhu Z., Li W., Fan C., Huang Q. (2011). Distribution and biocompatibility studies of graphene oxide in mice after intravenous administration. Carbon.

[28] Chong Y., Ma Y., Shen H., Tu X., Zhou X., Xu J., Dai J., Fan S., Zhang Z. (2014). The in vitro and in vivo toxicity of graphene quantum dots. Biomaterials.

[29] Su W.C., Ku B.K., Kulkarni P., Cheng Y.S. (2016). Deposition of graphene nanomaterial aerosols in human upper airways. J. Occup. Environ. Hyg..

[30] Sanchez V.C., Jachak A., Hurt R.H., Kane A.B. (2012). Biological interactions of graphene-family nanomaterials: An interdisciplinary review. Chem. Res. Toxicol..

[31] Jin H., Lai N., Jiang C., Wang M., Yao W., Han Y., Song W. (2025). Potential health risks of exposure to graphene and its derivatives: A review. Processes.

[32] Singh Z. (2016). Applications and toxicity of graphene family nanomaterials and their composites. Nanotechnol. Sci. Appl..

[33] Thakur N., Manna P., Das J. (2019). Synthesis and biomedical applications of nanoceria, a redox active nanoparticle. J. Nanobiotechnol..

[34] Bai Y., Li Y., Li Y., Tian L. (2024). Advanced Biological Applications of Cerium Oxide Nanozymes in Disease Related to Oxidative Damage. ACS Omega.

[35] Heckert E.G., Karakoti A.S., Seal S., Self W.T. (2008). The role of cerium redox state in the SOD mimetic activity of nanoceria. Biomaterials.

[36] Fu X., Li P., Chen X., Ma Y., Wang R., Ji W., Gu J., Sheng B., Wang Y., Zhang Z. (2024). Ceria nanoparticles: Biomedical applications and toxicity. J. Zhejiang Univ. Sci. B.

[37] Dahle J.T., Arai Y. (2015). Environmental geochemistry of cerium: Applications and toxicology of cerium oxide nanoparticles. Int. J. Environ. Res. Public Health.

[38] Montini T., Melchionna M., Monai M., Fornasiero P. (2016). Fundamentals and catalytic applications of CeO_2_-based materials. Chem. Rev..

[39] Zhou R., Gao H. (2014). Cytotoxicity of graphene: Recent advances and future perspective. Wiley Interdiscip. Rev. Nanomed. Nanobiotechnol..

[40] Corsi F., Deidda Tarquini G., Urbani M., Bejarano I., Traversa E., Ghibelli L. (2023). The impressive anti-inflammatory activity of cerium oxide nanoparticles: More than redox?. Nanomaterials.

[41] Tisi A., Pulcini F., Carozza G., Mattei V., Flati V., Passacantando M., Antognelli C., Maccarone R., Delle Monache S. (2022). Antioxidant properties of cerium oxide nanoparticles prevent retinal neovascular alterations in vitro and in vivo. Antioxidants.

[42] Ahamed M., Akhtar M.J., Khan M.A.M., Alaizeri Z.M., Alhadlaq H.A. (2019). Evaluation of the cytotoxicity and oxidative stress response of CeO_2_-RGO nanocomposites in human lung epithelial A549 cells. Nanomaterials.

[43] Al-Attar H.M., Mohammad M.H., Majeed A.M., Hussein H.T., Ahmed A.A. (2025). Investigating the anticancer activity of cerium nanoparticle decorated on GO produced by green methods against cancerous cell lines. Asian Pac. J. Cancer Prev..

[44] Tamtaji O.R., Ostadian A., Homayoonfal M., Nejati M., Mahjoubin-Tehran M., Nabavizadeh F., Ghelichi E., Mohammadzadeh B., Karimi M., Rahimian N. (2024). Cerium(IV) oxide:silver/graphene oxide (CeO_2_:Ag/GO) nanoparticles modulate gene expression and inhibit colorectal cancer cell growth: A pathway-centric therapeutic approach. Cancer Nano.

[45] Morgan D.J. (2023). Photoelectron spectroscopy of ceria: Reduction, quantification and the myth of the vacancy peak in XPS analysis. Surf. Interface Anal..

[46] Isaacs M.A., Drivas C., Lee R., Palgrave R., Parlett C.M.A., Morgan D.J. (2023). XPS surface analysis of ceria-based materials: Experimental methods and considerations. Appl. Surf. Sci. Adv..

[47] Lavkova J., Khalakhan I., Chundak M., Vorokhta M., Potin V., Matolin V., Matolinova I. (2015). Growth and composition of nanostructured and nanoporous cerium oxide thin films on graphite foil. Nanoscale.

[48] Škoda M., Cabala M., Matolínová I., Skála T., Veltruská K., Matolín V. (2009). A photoemission study of the ceria and Au-doped ceria/Cu(111) interfaces. Vacuum.

[49] Skoda M., Cabala M., Matolínová I., Prince K.C., Skála T., Sutara F., Veltruská K., Matolín V. (2009). Interaction of Au with CeO_2_(111): A photoemission study. J. Chem. Phys..

[50] Matolín V., Matolínová I., Sedlácek L., Prince K.C., Skála T. (2009). A resonant photoemission applied to cerium oxide based nanocrystals. Nanotechnology.

[51] Paparazzo E. (2011). On the curve-fitting of XPS Ce(3d) spectra of cerium oxides. Mater. Res. Bull..

[52] Ma L., Wang X., Wang J., Zhang J., Yin C., Fan L., Zhang D. (2021). Graphene oxide–cerium oxide hybrids for enhancement of mechanical properties and corrosion resistance of epoxy coatings. J. Mater. Sci..

[53] Magerusan L., Pogacean F., Pruneanu S. (2022). Enhanced acetaminophen electrochemical sensing based on nitrogen-doped graphene. Int. J. Mol. Sci..

[54] Bradshaw A.M., Cederbaum S.L., Domcke W., Krause U. (2014). Plasmon coupling to core hole excitations in carbon. J. Phys. C Solid State Phys..

[55] Anandan C., Bera P. (2013). XPS studies on the interaction of CeO_2_ with silicon in magnetron sputtered CeO_2_ thin films on Si and Si3N4 substrates. Appl. Surf. Sci..

[56] Bhakta-Guha D., Efferth T. (2015). Hormesis: Decoding two sides of the same coin. Pharmaceuticals.

[57] Janazsewska A., Banosz G. (2002). Assay of antioxidant capacity: Comparison of methods as applied to human blood plasma. Scand. J. Lab. Investig..

[58] Papanikolaou E., Simos Y.V., Spyrou K., Patila M., Alatzoglou C., Tsamis K., Vezyraki P., Stamatis H., Gournis D.P., Peschos D. (2023). Does Green Exfoliation of Graphene Produce More Biocompatible Structures?. Pharmaceutics.

[59] Zhang C., Zhang X., Zhang W., Zhao Z., Fan X. (2023). Functionalized graphene from electrochemical exfoliation of graphite toward improving lubrication function of base oil. Lubricants.

[60] Ejigu A., Kinloch I.A., Dryfe R.A. (2017). Single stage simultaneous electrochemical exfoliation and functionalization of graphene. ACS Appl. Mater. Interfaces.

[61] Liu X., Wu L., Yu X., Peng H., Xu S., Zhou Z. (2022). In-Situ Growth of Graphene Films to Improve Sensing Performances. Materials.

[62] Hofmann M., Chiang W.Y., Nguyễn T.D., Hsieh Y.P. (2015). Controlling the properties of graphene produced by electrochemical exfoliation. Nanotechnology.

[63] Pirmohamed T., Dowding S.M., Singh S., Wasserman B., Heckert E., Karakoti A.S., King J.E., Seal S., Self W.T. (2010). Nanoceria exhibit redox state-dependent catalase mimetic activity. Chem. Commun..

[64] Celardo I., Pedersen J., Traversa E., Ghibelli L. (2011). Pharmacological potential of cerium oxide nanoparticles. Nanoscale.

[65] Korsvik C., Patil S., Seal S., Self W.T. (2007). Superoxide dismutase mimetic properties exhibited by vacancy engineered ceria nanoparticles. Chem. Commun..

[66] Chen S., Wang Y., Bao S., Yao L., Fu X., Yu Y., Lyu H., Pang H., Guo S., Zhang H. (2024). Cerium oxide nanoparticles in wound care: A review of mechanisms and therapeutic applications. Front. Bioeng. Biotechnol..

[67] Mahdy H., Hendawy H., Abbas Y., Duraia E.-S. (2025). Electrospun CeO_2_-AgVO_3_/GO@PCL composite scaffolds: Structural, mechanical, and biological characterizations for advanced biomedical applications. BioNanoScience.

[68] Lasocka I., Jastrzębska E., Szulc-Dąbrowska L., Skibniewski M., Pasternak I., Kalbacova M.H., Skibniewska E.M. (2019). The effects of graphene and mesenchymal stem cells in cutaneous wound healing and their putative action mechanism. Int. J. Nanomed..

[69] Shin S.R., Aghaei-Ghareh-Bolagh B., Dang T.T., Topkaya S.N., Gao X., Yang S.Y., Jung S.M., Oh J.H., Dokmeci M.R., Tang X.S. (2013). Cell-laden microengineered and mechanically tunable hybrid hydrogels of gelatin and graphene oxide. Adv. Mater..

[70] Cha C., Shin S.R., Gao X., Annabi N., Dokmeci M.R., Tang X.S., Khademhosseini A. (2014). Controlling mechanical properties of cell-laden hydrogels by covalent incorporation of graphene oxide. Small.

[71] Zhang C., Lu T., Tao J., Wan G., Zhao H. (2016). Co-delivery of paclitaxel and indocyanine green by PEGylated graphene oxide: A potential integrated nanoplatform for tumor theranostics. RSC Adv..

[72] Thangamuthu M., Hsieh K.Y., Kumar P.V., Chen G.Y. (2019). Graphene- and graphene oxide-based nanocomposite platforms for electrochemical biosensing applications. Int. J. Mol. Sci..

[73] Krishnan S.K., Singh E., Singh P., Meyyappan M., Nalwa H.S. (2019). A review on graphene-based nanocomposites for electrochemical and fluorescent biosensors. RSC Adv..

[74] De S., Mohanty S., Nayak S.K. (2015). Nano-CeO_2_ decorated graphene based chitosan nanocomposites as enzymatic biosensing platform: Fabrication and cellular biocompatibility assessment. Bioprocess Biosyst. Eng..

[75] Ates G., Vanhaecke T., Rogiers V., Rodrigues R.M., Rodrigues R.M. (2017). Assaying cellular viability using the neutral red uptake assay. Methods in Molecular Biology.

[76] Repetto G., del Peso A., Zurita J.L. (2008). Neutral red uptake assay for the estimation of cell viability/cytotoxicity. Nat. Protoc..

