# Supplementary material for: Structure–Biomedical Activity Relationship of Tunable Ceria–Graphene Nanocomposites Leading to Divergent Cellular Responses
Source: Int J Mol Sci. 2026 May 26;27(11):4772. doi: 10.3390/ijms27114772 (PMC13256777; doi:10.3390/ijms27114772)
Supplement: Supplementary file 1 [file ijms-27-04772-s001.zip › ijms-4208520-supplementary.pdf]

Electronic Supplementary Materials

Structure–Biomedical Activity Relationship of Tunable Ceria–Graphene Nanocomposites Leading to Divergent Cellular Responses

Tudor-Mihai Magdaş [1,2,3], Ioana Bâldea [4], Constantin Bodolea [1,5], Andrei Mihai Bălan [1,5], Adrian Ștef [1,3], Lidia Măgeruşan [2,*] and Gabriela Adriana Filip [2,6]

1 Anesthesia and Intensive Care Department, "Iuliu Hatieganu" University of Medicine and Pharmacy, 8 Victor Babeş Street, 400012 Cluj-Napoca, Romania; tudor.miha.magdas@elearn.umfcluj.ro (T.-M.M.); constantin.bodolea@umfcluj.ro (C.B.); balan.andrei@umfcluj.ro (A.M.B.); stef.adrian@yahoo.com (A.Ș.)

2 National Institute for Research and Development of Isotopic and Molecular Technologies, 67-103 Donat Street, 400293 Cluj-Napoca, Romania; gabriela.filip@umfcluj.ro

3 Department of Anesthesia and Intensive Care, Heart Institute "Niculae Stancioiu", "Iuliu Hatieganu" University of Medicine and Pharmacy, 19-21 Motilor Street, 400001 Cluj-Napoca, Romania

4 Department of Physiology, Faculty of Medicine, University of Medicine and Pharmacy "Iuliu Hatieganu", 400347 Cluj-Napoca, Romania; ioana.baldea@umfcluj.ro

5 Department of Anesthesia and Intensive Care, Municipal Clinical Hospital, 400139 Cluj-Napoca, Romania

6 Department of Anatomy and Embryology, "Iuliu Hațieganu" University of Medicine and Pharmacy, 8 Victor Babes Street, 400012 Cluj-Napoca, Romania

* Correspondence: lidia.magerusan@itim-cj.ro

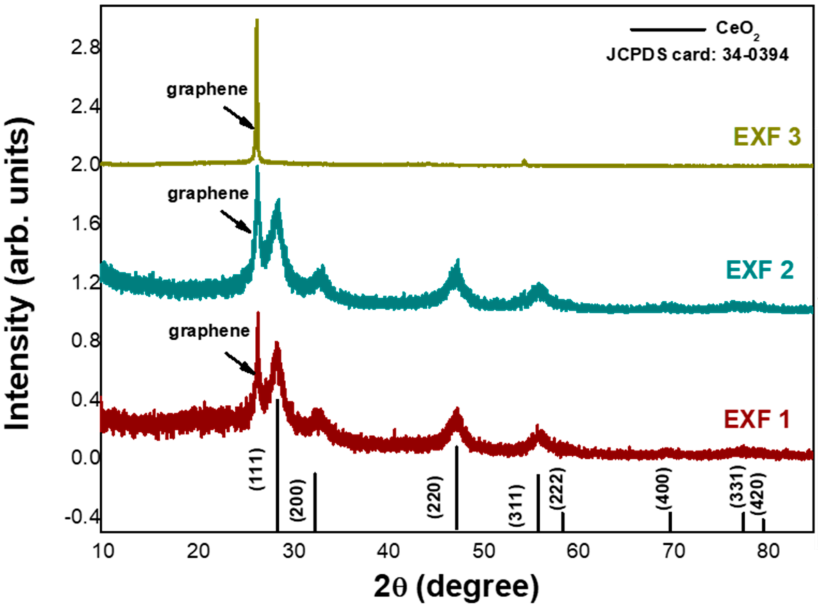

Figure S1. XRD patterns corresponding to EXF 1, EXF 2 and EXF 3 and the identification of main diffraction lines corresponding to CeO_2 structure

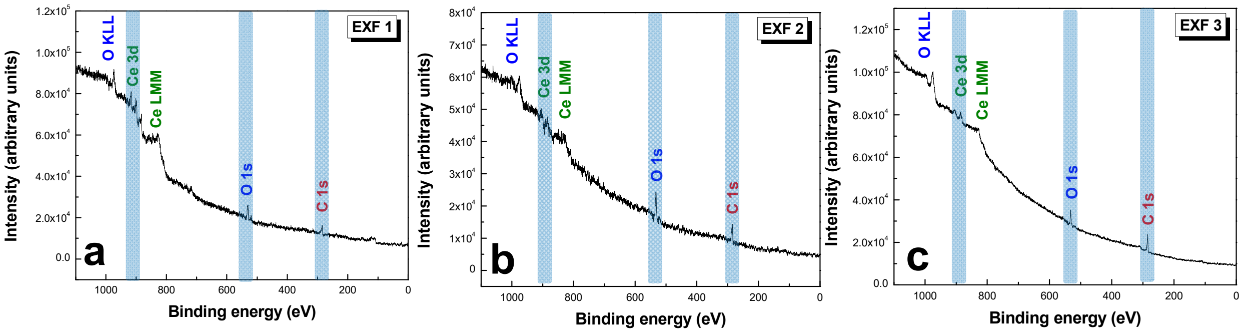

FigureS2. XPS survey spectra corresponding to EXF 1(a), EXF 2 (b) and EXF 3 (c)

Table S1. The atomic concentrations of the detected elements calculated from the corresponding XPS survey spectra

Sample	C (at%)	O (at %)	Ce (at %)
**EXF 1**	48.72	44.12	7.15
**EXF 2**	64.70	29.17	6.14
**EXF 3**	73.56	25.10	1.34

Table S2. Binding energies (BE) and atomic concentrations [AC, %] assigned for each deconvolution component used in the *Ce 3d*, *C1s* and *O1s* high resolution XPS spectra fitting corresponding to EXF1, EXF 2 and EXF 3

<table>
<tr><th>Sample</th><th colspan="16"></th></tr>
<tr><th></th><th>Element</th><th colspan="15">Binding energy (eV) / Assignment [AC, %]</th></tr>
<tr><td rowspan="7">EXF1</td><td rowspan="5">Ce</td><td colspan="15">Ce^{4+}</td></tr>
<tr><td colspan="3">882.74
[26.57 %]</td><td colspan="3">885.92
[9.01 %]</td><td colspan="3">903.22
[8.53 %]</td><td colspan="3">907.32
[5.30 %]</td><td colspan="3">916.93
[11.92 %]</td></tr>
<tr><td colspan="15">Ce^{3+}</td></tr>
<tr><td colspan="5">888.88
[12.85 %]</td><td colspan="5">898.63
[18.51 %]</td><td colspan="5">901.14
[7.31 %]</td></tr>
<tr></tr>
<tr><td>C</td><td colspan="7">284.43 eV [43.70 %]
285.42 eV [19.18 %]
286.24 eV [9.49 %]
287.14 eV [7.87 %]
289.02 eV [15.74 %]
291.72 eV [4.02 %]</td><td colspan="8">sp² C=C / CH_n
sp³ C-C / CH_n
C-O
C=O
OH-C=O / COOH
$\pi \rightarrow \pi^*$ shake up satellite</td></tr>
<tr><td>O</td><td colspan="7">529.59 [42.57 %]
531. 09 [27.14 %]
532.47 [26.27 %]
534.32 [4.03 %]</td><td colspan="8">Ce-O
O-H
C=O
C-O</td></tr>
<tr><td rowspan="5">EXF2</td><td rowspan="4">Ce</td><td colspan="15">Ce^{4+}</td></tr>
<tr><td colspan="3">882.63
[18.91 %]</td><td colspan="3">885.56
[18.92 %]</td><td colspan="3">904.01
[12.41 %]</td><td colspan="3">906.65
[11.93 %]</td><td colspan="3">917.18
[4.63 %]</td></tr>
<tr><td colspan="15">Ce^{3+}</td></tr>
<tr><td colspan="5">887.70
[14.15 %]</td><td colspan="5">899.34
[12.28 %]</td><td colspan="5">901.65
[6.77 %]</td></tr>
<tr><td>C</td><td colspan="7">284.41 eV [43.13 %]
285.58 eV [17.71 %]
286.62 eV [18.54 %]
287.89 eV [9.06 %]
289.22 eV [8.07 %]
291.57 eV [3.48 %]</td><td colspan="8">sp² C=C / CH_n
sp³ C-C / CH_n
C-O
C=O
OH-C=O / COOH
$\pi \rightarrow \pi^*$ shake up satellite</td></tr>
</table>

<table>
<tr><td></td><td>O</td><td colspan="15">529.20 [44.05 %]
531. 62 [35.01 %]
532.10 [15.59 %]
534.53 [5.35 %]</td><td colspan="15">Ce-O
O-H
C=O
C-O</td></tr>
<tr><td rowspan="7">EXF3</td><td rowspan="4">Ce</td><td colspan="30">Ce^{4+}</td></tr>
<tr><td colspan="6">882.28
[17.10 %]</td><td colspan="6">885.11
[24.36 %]</td><td colspan="6">905.59
[5.92 %]</td><td colspan="6">907.75
[4.51 %]</td><td colspan="6">917.77
[4.29 %]</td></tr>
<tr><td colspan="30">Ce^{3+}</td></tr>
<tr><td colspan="10">887.44
[14.75 %]</td><td colspan="10">900.06
[10.77 %]</td><td colspan="10">903.37
[18.30 %]</td></tr>
<tr><td>C</td><td colspan="15">284.29 eV [40.34 %]
284.86 eV [26.79 %]
286.42 eV [20.59 %]
287.93 eV [3.69 %]
288.79 eV [5.88 %]
291.03 eV [2.71 %]</td><td colspan="15">sp^2 C=C / CH_n
sp^3 C-C / CH_n
C-O
C=O
OH-C=O / COOH
$\pi \rightarrow \pi^*$ shake up satellite</td></tr>
<tr><td>O</td><td colspan="15">531.09 [23.19 %]
532. 18 [53.29 %]
533.27 [17.72 %]
534.87 [5.80 %]</td><td colspan="15">Ce-O
O-H
C=O
C-O</td></tr>
</table>

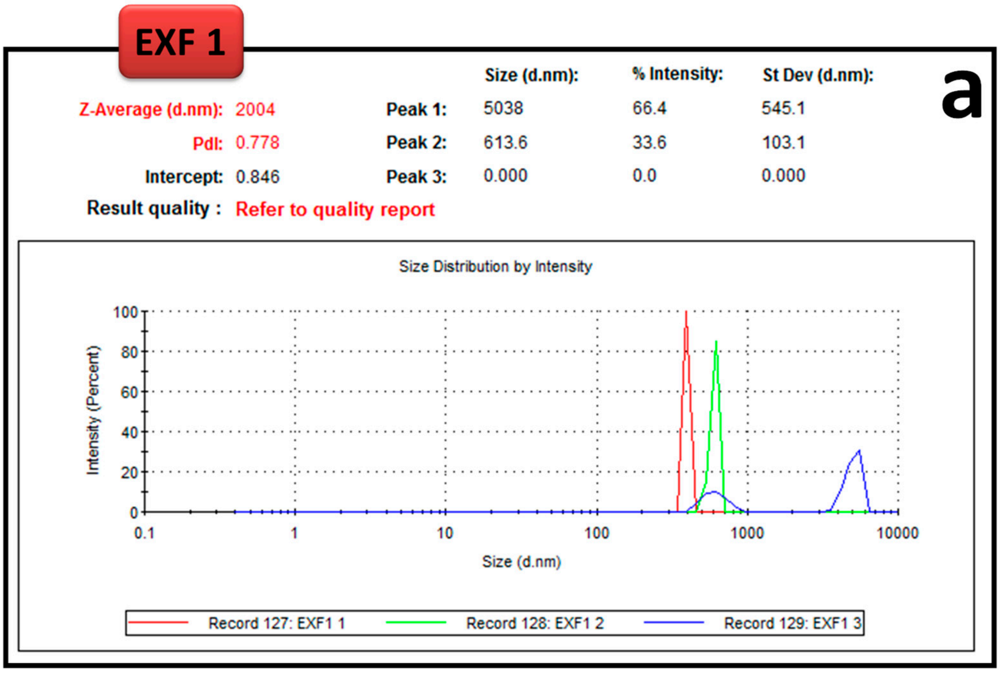

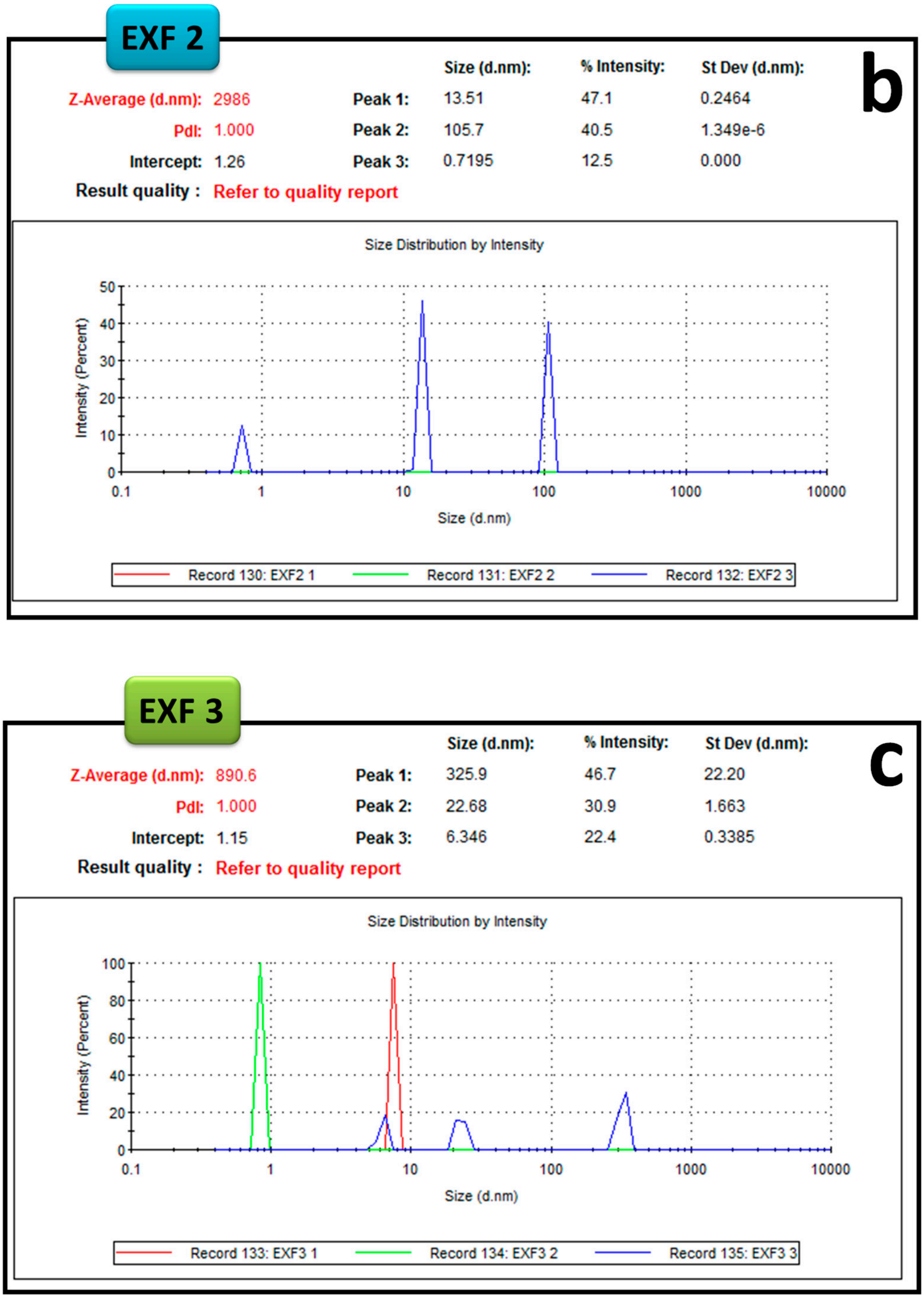

FigureS3. Dynamic Light Scattering measurements corresponding to EXF 1(a), EXF 2 (b) and EXF 3 (c)

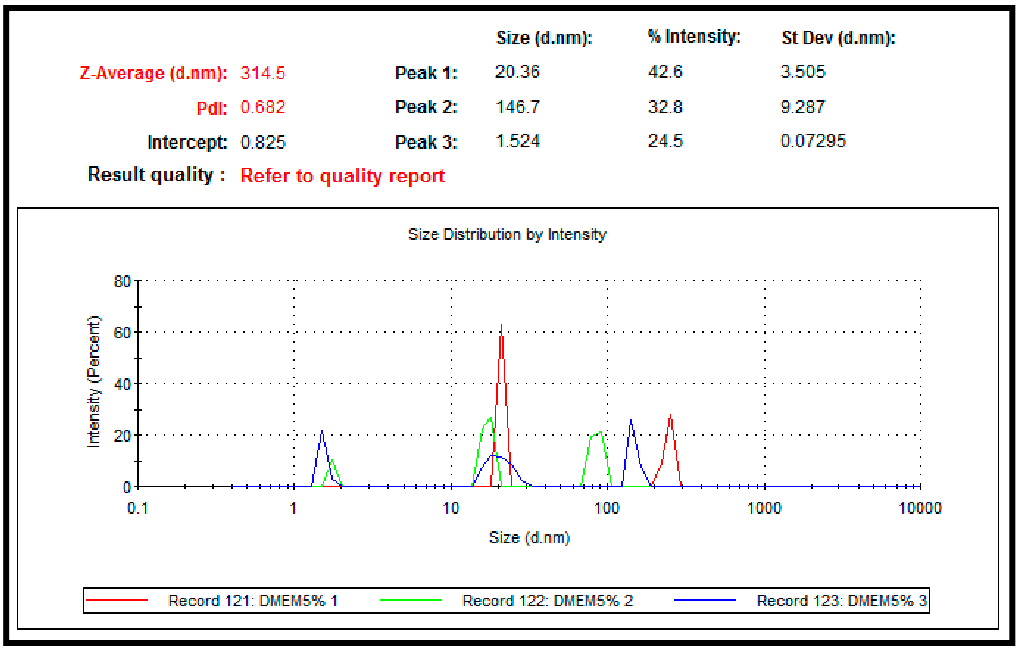

Figure S4. Dynamic Light Scattering measurements corresponding to culture medium

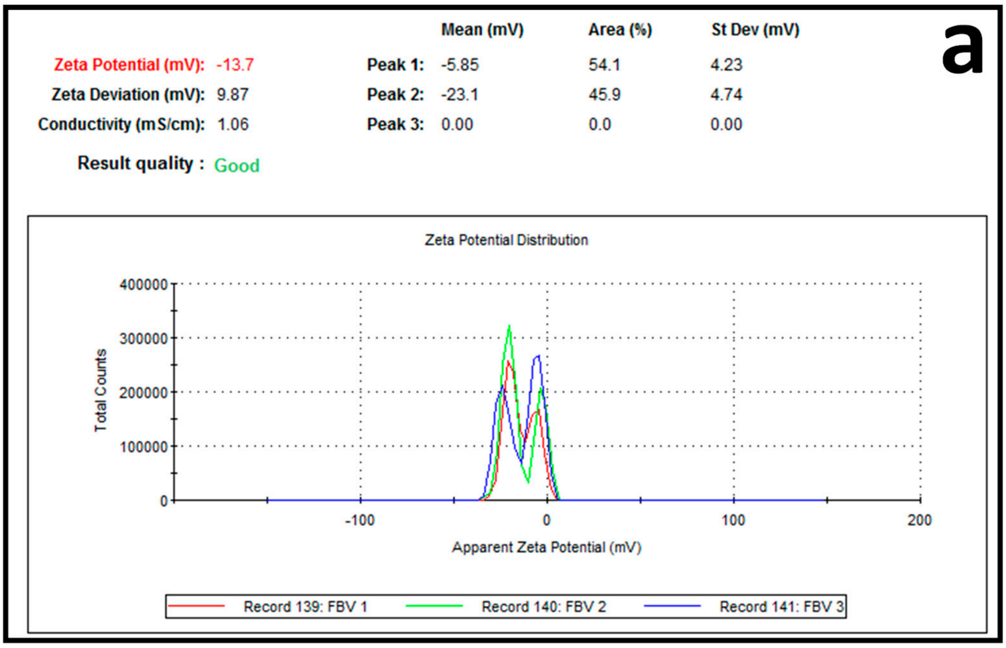

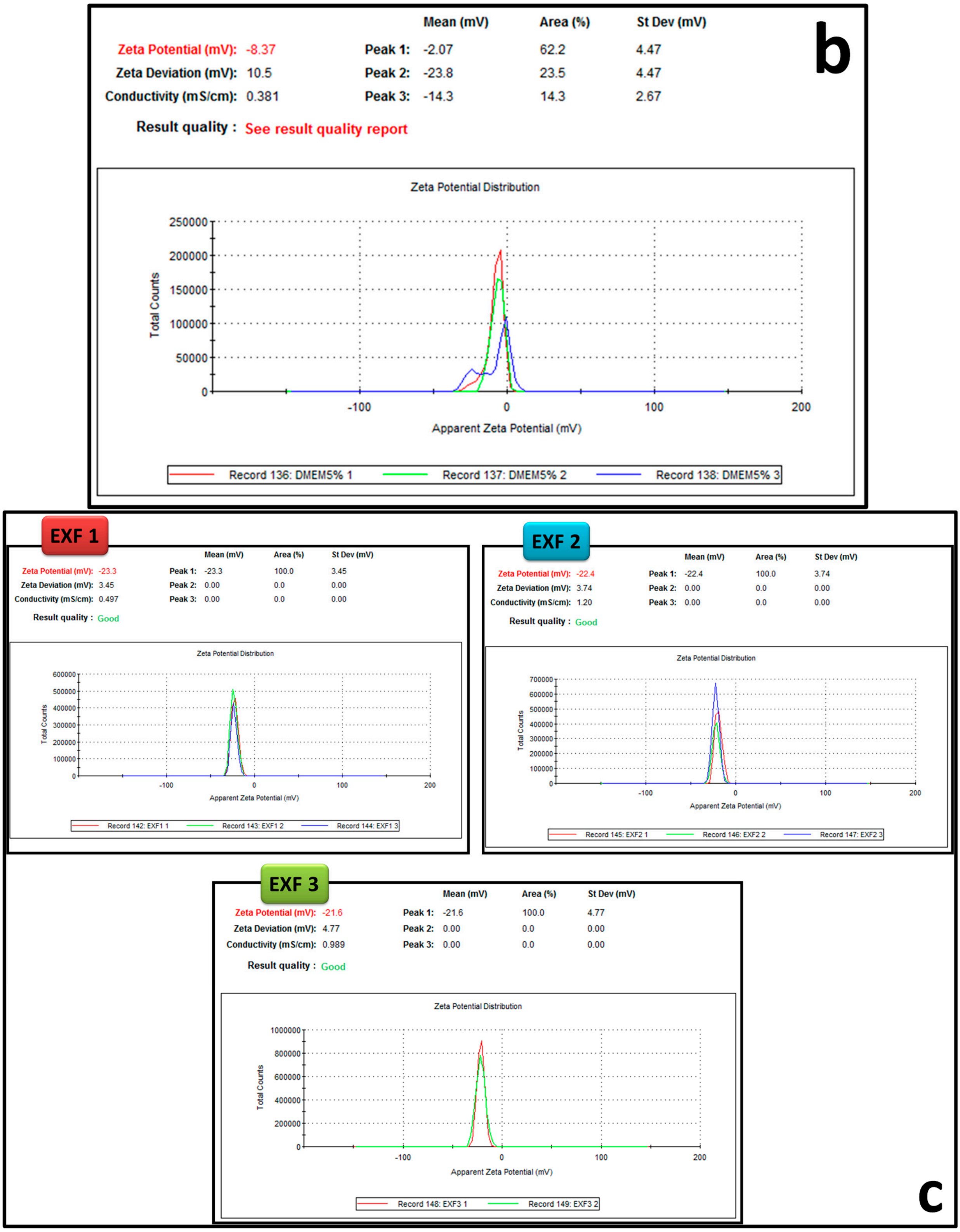

Figure S5. Zeta potential results corresponding to bovine serum (a) culture medium (b), in presence of EXF1, EXF 2 and EXF 3 (c)
